# Editorial: G Protein-Coupled Receptor Kinases (GRKs) and β-Arrestins: New Insights Into Disease Regulators

**DOI:** 10.3389/fphar.2019.01654

**Published:** 2020-01-28

**Authors:** Yuichi Hattori, Martin C. Michel

**Affiliations:** ^1^ The Research Institute of Cancer Prevention, Health Sciences University of Hokkaido, Tobetsu, Japan; ^2^ Department of Pharmacology, Johannes Gutenberg University, Mainz, Germany

**Keywords:** G protein-coupled receptor, G protein-coupled receceptor kinase, arrestin, heart, cancer

G protein-coupled receptors (GPCRs) are the largest family of plasma membrane proteins mediating cellular responses to a wide variety of external stimuli and can be crucially involved in a multitude of physiological process and their dysregulation contributes to many diseases. G protein-coupled receptor kinases (GRKs) and β-arrestins were initially identified as a pivotal player in the process of desensitization of agonist-activated GPCRs (Premont and Gainetdinov, 2007; Black et al., 2016): GRKs specifically phosphorylate agonist-activated GPCRs, and receptor phosphorylation triggers the binding of cytoplasmic β-arrestin molecules, which sterically block the activation of heterotrimeric G proteins, leading to rapid desensitization of G protein-mediated signaling cascades. However, growing evidence suggests GRKs and β-arrestins fulfill a vital role in regulating a variety of cellular proteins involved in signal transduction independently of GPCRs (Penela et al., 2010). Thus, GRKs and β-arrestins can interact with non-GPCRs. GRKs and β-arrestins may directly affect functioning of non-GPCRs or indirectly regulate non-GPCR signaling. In addition, incoming evidence supports that changes in function and/or expression of GRKs and β-arrestins may be important in cardiovascular, inflammatory, metabolic, or cancer pathologies (Vroon et al., 2006; Schumacher and Koch, 2017; Steury et al., 2018; Yu et al., 2018). A better understanding of the pathological roles of GRKs and β-arrestins would provide a basis for new therapeutic targets in different human diseases.

We organized the Research Topic entitled “G protein-coupled receptor kinases (GRKs) and β-arrestins: new insights into disease regulators” in *Frontiers in Pharmacology*, which started at February, 2018. A total of 12 articles, consisting of 3 original papers and 9 review papers, has been published in *Frontiers in Pharmacology*. Our Research Topic has been well received by the readership of the journal with about 20,000 views.

In this Research Topic, Sun et al. advocated the unconventional role of β-arrestin 2 in promoting hepatocyte apoptosis in alcoholic liver disease. It is documented that apoptosis of massive hepatocytes is a prominent feature of the initiation and progression stages of alcoholic liver disease (Ceni et al., 2014). Sun et al. demonstrated that β-arrestin 2 levels in liver tissues from ethanol-fed mice were markedly higher than those from control diet-fed mice and knockdown of β-arrestin 2 inhibited hepatocyte apoptosis induced by ethanol *in vivo*. As deficiency of β-arrestin 2 increased phosphorylation of Akt and overexpression of β-arrestin 2 suppressed Akt activation in AML-12 mouse hepatic cell line which exhibited a reduction in Akt phosphorylated levels in response to ethanol exposure, β-arrestin 2 appears to promote hepatocyte apoptosis through suppression of the cell survival regulator Akt.

Palikhe et al. have demonstrated that GRK2 can function as Toll-like receptor (TLR) signaling to elicit inducible nitric oxide synthase (iNOS) expression in mouse MG6 microglial cells. They revealed that GRK2 strongly regulated the expression/activation of IRF1as well as the activation of the STAT pathway, leading to increased transcription of iNOS, when TLR-3, TLR-4, or TLR-9 was stimulated in microglial cells. This extends their previous report showing that GRK2 plays a critical role in iNOS gene transcription in microglial cells stimulated with lipopolysaccharide (Kawakami et al., 2018). Their findings highlight a novel pathological role of GRK2 in regulating inflammatory signaling in microglia with a potential therapeutic window for some neuroinflammatory disorders.

Cannavo et al. have identified GRK2 as a relevant player in the aldosterone signaling pathway. Aldosterone is produced not only in adrenals but also in cardiovascular tissues, and has been implicated in the development of cardiac hypertrophy and fibrosis (Jewell et al., 2006). Cannavo et al. (2016) have previously reported that GRK2 and GRK5 lie downstream of the aldosterone/mineralocorticoid receptor system. In this study, using *in vivo* two mouse models of hyperaldosteronism, chronic aldosterone infusion and surgical myocardial infarction, and *in vitro* 3T3-L1 fibroblast cultures, they demonstrated that canonical and noncanonical actions of GRK2 are involved in aldosterone-triggered attenuation of insulin- and β-adrenoceptor-mediated effects at the heart levels. Their finding that spironolactone, a mineralocorticoid receptor antagonist, offset cardiac insulin-signaling dysfunction and β_1_-adrenoceptor downregulation in mice with myocardial infarction by blunting canonical and noncanonical effects mediated by GRK2 levels may provide a novel mechanism for the cardioprotective effect of aldosterone antagonists.

Several review articles highlighted the novel roles of GRKs and β-arrestins as crucial modulators in the pathogenesis of a variety of diseases. Murga et al. summarized the pathophysiological roles of GRK2 in cardiovascular and metabolic diseases, such as heart failure, hypertension, obesity and insulin resistance conditions, and non-alcoholic fatty liver disease (NAFLD). Furthermore, they discussed different strategies to target GRK2 functionality as a potentially relevant approach to treat cardiovascular disease, obesity, type 2 diabetes, or NAFLD. Mangmool et al. summarized evidence that GRKs and β-arrestins could be potential candidates for novel therapeutic strategies for heart failure, including the view that carvedilol, alprenolol, and nebivolol are identified as β-arrestin-biased β-blockers, based on the roles of GRKs and β-arrestins on how they affect cardiac β-adrenoceptor signaling regarding the molecular and cellular pathophysiology. Bagnato and Rosanò highlighted the role of GPCR/β-arrestins-dependent signal pathways in cancer growth, invasion, and metastasis, although it remains to be learned both about how β-arrestins mediate gene expression changes to execute the GPCR-induced pro-metastatic effects in tumor cells and about how β-arrestins and G protein-mediated effects may differ in this regard. Bond et al. summarized that β-arrestins are involved in the pathophysiology of numerous and wide-ranging diseases, including asthma and cancer, and described the mechanisms by which β-arrestins regulate GPCR signaling, including the functional cellular mechanisms modulated by β-arrestins and related this to observed pathophysiological responses associated with β-arrestins.

Recent technological advancements in molecular and structural biology have provided new insights into the roles of GRKs and β-arrestins in not only GPCR-dependent but also non-GPCR-mediated signaling mechanisms. Given that GRKs and β-arrestins are important in signaling pathways and processes related to a variety of disease conditions, they could be expected as a promising therapeutic target in some diseases. A number of small molecules, peptides, and inhibitory constructs are being developed to target GRKs and β-arrestins, but we face the issues that need to be addressed in this drug discovery in light of the complexity of GPCR signaling pathways and the pleitropy of GRK and β-arrestin functions. However, the unwavering search for modulators targeting GRKs and β-arrestins will bestow a great impact on future therapies for a variety of nasty diseases.

## Author Contributions

YH and MM initiated the Research Topic. YH drafted the editorial and MM revised it for critical content. YH and MM have read and approved the final manuscript and take full responsibility for it.

## Funding

Work on GPCR in the lab of YH is funded by Grants-in-Aid for Scientific Research from Japan Society for Promotion of Science (26460336, 17K08586). That in the lab of MCM is funded by the Deutsche Forschungsgemeinschaft (Mi 294/10-1).

## Conflict of Interest

The authors declare that the research was conducted in the absence of any commercial or financial relationships that could be construed as a potential conflict of interest.
